# Use of lumen-apposing metal stents (LAMS) in the management of gastro jejunostomy stricture following Roux-en-Y Gastric Bypass for obesity: a prospective series

**DOI:** 10.1186/s12893-021-01310-3

**Published:** 2021-07-17

**Authors:** Adam Peter Skidmore

**Affiliations:** 1 Victorian Obesity Surgery Centre, Suite1/Level 10 Martin St, Heidelberg, VIC 3084 Australia; 2 Warringal Private Hospital, Heidelberg, Australia; 3 Albury Wodonga Health and Albury Wodonga Private Hospital, Albury, NSW Australia

**Keywords:** Roux-en-Y Gastric Bypass, Obesity, Obesity, Endoscopy, Esophageal stents, Anastomotic stricture

## Abstract

**Background:**

Chronic strictures following Roux-en-Y Gastric Bypass (RYGB) are a troublesome complication that can lead to significant morbidity. The use of stents has been described but the need for X-ray and risk of migration have meant limited use in the management of these strictures. Lumen apposing metal stents (LAMS) have traditionally been used for management of pancreatic pseudocysts. They don’t require X-ray and are easy to deploy with a short learning curve. This paper explores the use of LAMS to treat post RYGB strictures and explores their safety and efficacy.

**Methods:**

A prospective study over a 4-year period looking at 14 patients with post RYGB strictures. These patients were privately insured patients operated within a tertiary Private facility. The patients were followed up for between 1 and 3 years. We have prospectively collected data on the efficacy and safety of LAMS in these patients. Patients were followed up until stent removal or definitive surgery to correct a stricture.

**Results:**

421 patients underwent RYGB in the study period. 14 (3.3%) of these patients developed a stricture that resulted in insertion of LAMS. There was no immediate complications and 12 patients had complete resolution of their stricture. There were no reoperations due to migration related issues although a migration rate of 19% was noted. 2 patients required surgery to correct refractory strictures not relieved by a LAMS stent, both of these were strictures associated with marginal ulceration of the gastro jejunostomy.

**Conclusion:**

LAMS are a safe and effective method to manage post RYGB strictures. They have a high rate of resolution of strictures and can be safely deployed across strictures with no immediate complication. Migration does still appear to be a problem, however, does not appear to affect patient outcome or increase morbidity. Insertion is straightforward and doesn’t appear to be associated with a long learning curve.

## Background

Obesity is a major issue facing modern society. The prevalence has increased dramatically in recent years. In the United States, approximately 1 in 5 individuals over the age of 18 years have a body mass index (BMI) > 30 [1].

Roux-en-Y gastric bypass (RYGB) is a safe and effective method of producing sustained and meaningful weight loss, improvement or resolution of obesity-associated co-morbidities [2]. Anastomotic strictures are a known complication of the Gastro jejunostomy. Strictures have been reported to occur in 3–27% of RYGB’s [3]. Treatment has varied from endoscopic therapy to operative revision of the gastrojejunostomy with or without additional anatomic revisions [4]. Long term success with dilatation and stenting is still only achieved in 23–40% of patients [5]. To date we don’t believe there has been data specifically relating to use of the AXIOS™ stent and RYGB.

We present a novel technique of using the AXIOS™ stent (Boston Scientific). A Lumen apposing metal stent (LAMS) to keep the anastomosis open for up to 106 days post stricture. A prospective series of patients is presented. The aim being safe placement of LAMS to avoid multiple endoscopic interventions and ultimately increase the chance of resolution of strictures without multiple dilatations.

## Methods

A prospective audit of a single surgeon’s practice at Warringal and Albury Private Hospitals was established. A retrospective evaluation of the data was performed specifically focused on RYGB and strictures. Full informed consent was obtained from patients prior to placement on the register. Ethical approval was obtained for this study at the ethics committee of the Warringal Hospital and Albury private hospital. All procedures were performed by one experienced bariatric surgeon (> 950 cases). There were no additional exclusion criteria. All prospectively collected data were placed into a computerized research database starting from January 2014. In case of missing information, a detailed audit of all patients undergoing RYGB was once more retrospectively reviewed.

A standard technique was used for the gastrojejunostomy. A 45 mm Ethicon stapler was used to anastomose the gastric pouch to the Jejunum. The stapler was introduced to pouch via small gastrotomy and the jejunum through a small enterotomy. The anvil was introduced to 30 mm and fired to create a side to side posterior back wall stapled anastomosis. The resultant anterior defect was closed over a 32 French Visi G bougie. A 3′0 Monocryl V Lock was used to close the anterior defect. A methylene blue leak test was always performed via the Visi G Bougie.

A stricture was suspected when early regurgitation, reflux or vomiting was noted. An urgent early gastroscopy was performed by the operating surgeon. Once stricture was proven a decision to dilate with a 15 mm CRBE balloon or place LAMS was made. If the scope could not be passed through the stricture and the stricture was less than 1.5 cm’s in length a LAMS was placed without dilatation. Initially all stents were left for a period of 4–6 weeks. Some patients required multiple stents. This study observes safety, efficacy in the short and long term and adverse outcomes. We also identify factors that may delay resolution of strictures.

### Endoscopic technique

**Fig. 1:**
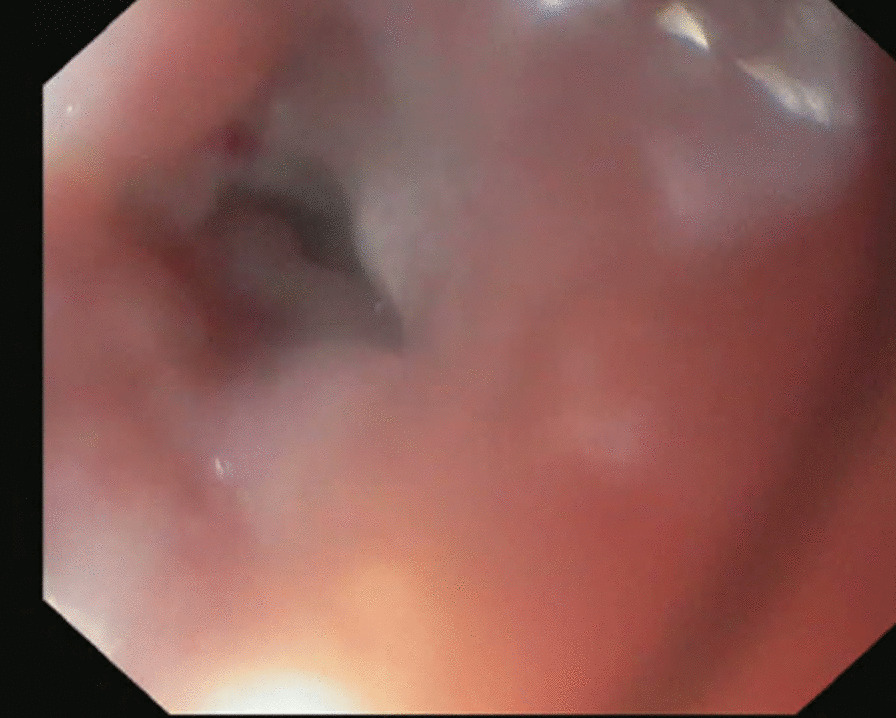
Pre stent deployment

LAMS are designed for deployment through a therapeutic forward viewing endoscope [6]. The technique used was similar to deployment used in pancreatic pseudocysts [7]. In this series a 15 mm stent was deployed. In most cases the endoscopist directly visualises the stricture (Fig. 1) and can assess the size and length. In some instances, the lumen can’t be traversed by the endoscope and the stent was placed after introduction of a guide wire. Radiology was not required in any of the reported cases. Stricture size was documented by the endoscopist and recorded for analysis.

**Fig. 2:**
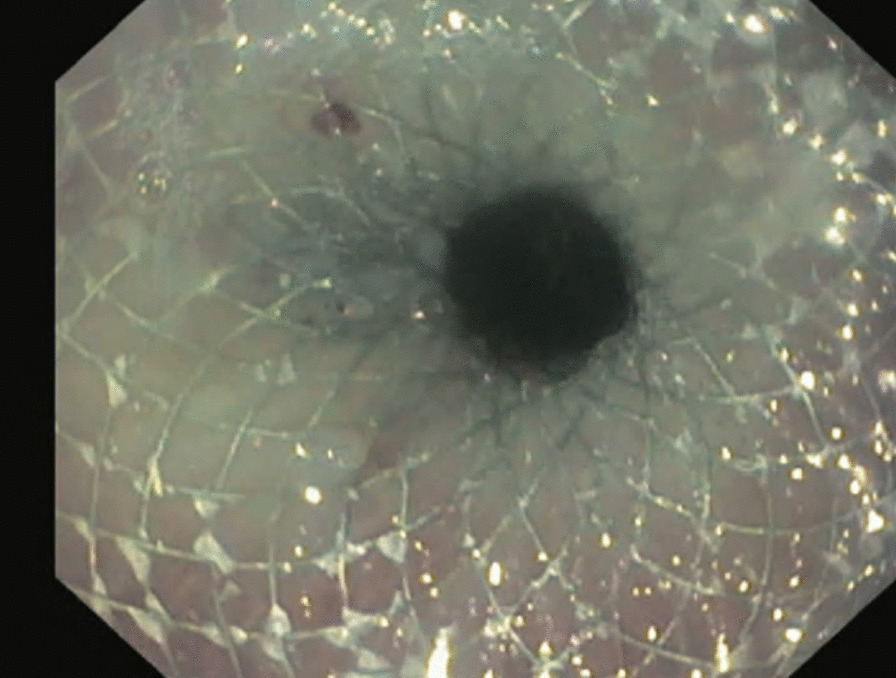
Immediate post stent deployment

**Fig. 3.**
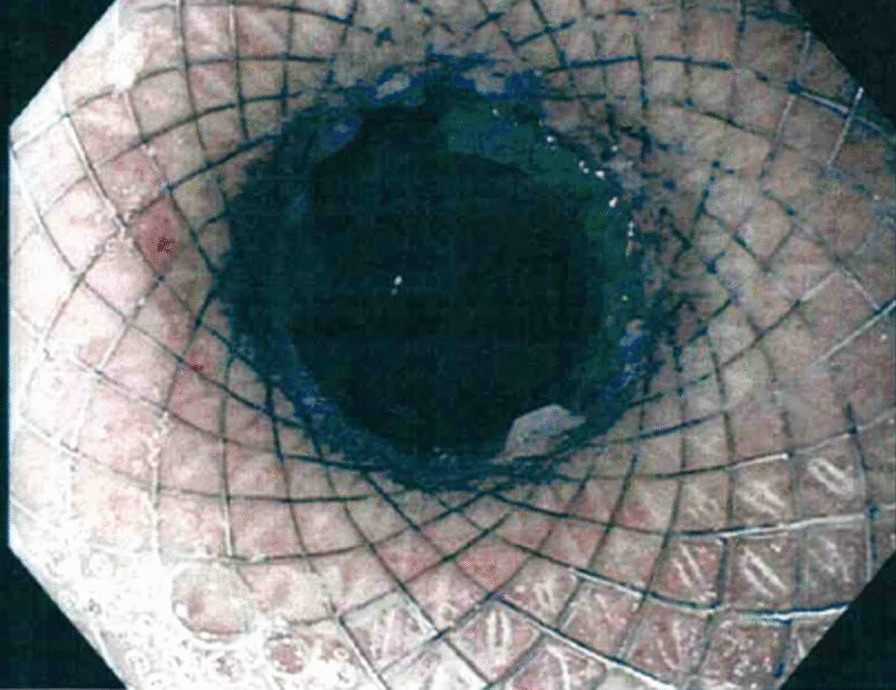
6 weeks post stent deployment

Pre stent dilatation was not performed to lessen the risk of stent migration. In all cases a short Jagwire™ was passed through the stricture to facilitate placement of the stent. The stent was then placed over the wire and the distal flange of the stent was then deployed and pulled back to allow deployment of the proximal flange. The proximal flange is deployed separately above the shoulder of the stricture. The stent was not traversed by the gastroscope at conclusion of deployment however jejunal mucosa could be observed beyond the stent and a distal lumen observed (Figs. 1, 2, 3).

## Results

14 Patients out of a total of 421 patients (3%) undergoing RYGB presented with a post-operative gastro jejunostomy stricture requiring dilatation. 12 of these patients presented within the first 6 weeks. One patient presented at Day 3 with sudden onset of dysphagia. 2 patients had strictures as a consequence of marginal ulcers.

These 14 patients all underwent insertion of a LAMS stent in preference to dilatation alone. On average the stent remained in for 44 days (10–161 days) until removal. A total of 26 stents were placed in these 14 patients (Table 1).

**Table 1:** Patient details and outcomes with LAMS

Patient	Time since stent removed (Months)	Weight at surgery (KG)	Weight at 3 months (KG)	Weight at 6 months (KG)	Current weight (KG)	TBWL	Previous surgery	Further surgery post stent
1	37	96	85	75	72	25%	LSG	No
2	35	191	181	166	135	31%	Gastric stapling	No
3	34	116	98	86	77	33%	LSG	No
4	22	112	94	80	67	40%	Gastric band	No
5	16	130	83	61	75	42%	Gastric band/LSG	Merindino 11/19
6	Stent in situ	127	104	87	99	22%	LSG stricture	No (overstitch suture removed)
7	12	138	112	98	50	63%	Primary RYGB	No
8	13	117	88	74	78	33%	LSG with stricture	No
9	15	120	104	88	68	43%	Gastric band	No
10	12	124	110	95	88	29%	LSG	Hiatal hernia repair
11	14	111	101	91	89	20%	RYGB with large pouch	No
12	Stent in situ	160	140	116	68	57%	None	Revision gastrojejunostomy
13	9	120	103	84	70	41%	LSG with stricture	No
14	7	98	87	68	57	42%	LSG with stricture	No
Total						37%		

Endoscopic assessment of the diameter of the stricture is difficult however the size was estimated by an experienced senior surgeon (the primary author). The average estimated stricture diameter was 7 mm (3–11 mm).

Five patients required re insertion of their stent. Two of these patients went on to have a formal revision of their Gastro jejunostomy. The remaining three patients strictures resolved after removal of the second stent.

The 2 patients who required revision of their gastro jejunostomy were not typical strictures. Both had a non-healing Marginal Ulcer that were refractory to medical treatment **(**patient 5 and 12, Table 1). Despite maximal medical treatment and after 2 stents we elected to resect the ulcer and in one instance reverse the bypass as a Merindino procedure. This was partially because of hypoalbuminemia and chronic diarrhoea. This patient was unable to tolerate any high protein food without the stent. The second stent was placed to enable improvement of her nutritional status prior to surgery.

Another patient (Patient 6/Table 1) had an overstitch procedure to narrow the gastro jejunostomy and developed a refractory stricture. Initially the stent was placed to encourage erosion of the Prolene suture. This would allow removal of the suture and dilatation. After multiple stents we have now been able to remove significant amounts of the suture and the patient is asymptomatic.

Patient 7 (Table 1) had a high-grade stricture which presented 2 days post operatively. She required 4 stents to get resolution of the stricture but is now asymptomatic with an excellent endoscopic result.

5 out of the 26 stents placed (19%) migrated distally and passed spontaneously. This was proven by repeat endoscopy and abdominal X-ray or CT scan. None of these required any intervention to remove them after migration.

There were no immediate or late complications after insertion of LAMS. Two patients had removal of the stent secondary to chest pain. In one of these patients the stent had migrated proximally in a short pouch to straddle the Gastroesophageal junction.

There were no immediate complications such as bleeding or ulceration.

Weight loss did not seem to be significantly affected by insertion of the stent. It appears to be comparable with patients on the data base with no evidence of stricture or insertion of stent (Tables 1 and 2).

**Table 2:** Average weight loss details of patients without stricture

Patients with no stricture	Average BMI pre-operative	Revision/primary	Average weight at surgery	Average TBWL
377	44	286 Rev/ 91 Primary	131 kg	34%

## Discussion

Roux-en-Y gastric bypass procedure is an effective treatment for morbid obesity. One of the most frequent complications after this operation is the appearance of a gastrojejunal anastomotic stricture [8]. Treatment has traditionally been dilatation with either pneumatic balloon or Savoury Gillard Bougie’s.

Previous studies have shown up to three dilatations can be needed to achieve symptomatic relief [1]. It is postulated that Linear stapled anastomosis are less likely to present with post-operative stricture compared to Circular stapler [9]. Nonetheless stricture rates of up to 6% have been described with this technique [10]. Gastro jejunostomy strictures can pose a difficult complication to deal with and can increase costs to the health care system.

LAMS have been traditionally used for management of pancreatic pseudocysts [11], however they have been increasingly used for other Upper gastro intestinal luminal strictures [6]. They have also been used for low rectal strictures with some success [12]. Longer self-expanding metal stents (SEMS) traditionally used for malignant strictures have been used for management of post RYGB leaks [13]^14^ but not frequently for anastomotic strictures. These stents require X-ray and have a learning curve associated with placement. They often require fixation to prevent migration which can result in significant morbidity to the patient.

LAMS with endoscopic suture for stricture resolution has been described [15]. In this series suture of the stent with Overstitch™ was used to anchor the stent. This series was a smaller series with 9 patients and only 6 patients in this series had undergone RYGB. They reported no migrations in their series, however they comment on the technical difficulty of endoscopic suture. Migration still tends to be problematic in our series with a rate of 19%. Despite this no major morbidity with these migrations was observed. Another group observed a 7% migration rate and a 21% failure rate when LAMS were used for benign upper GI strictures [16].

A concern with SEMS is the risk of migration both distally and proximally. This can result in subsequent need for removal of these stents. Migration rates of up to 52% have been described [17]. This may even be higher without suturing the stent in. This increases the complexity of using stents as it generally involves a concurrent laparoscopy. Endoscopic clips can be unreliable. Overstitch™ fixation of a LAMS as described by Simsek et al. [15] may be an alternative but is technically difficult to use with the LAMS geometry. Once the stricture has resolved SEMS tend to slip through the area and migrate distally. Tissue ingrowth can also be an issue with SEMS. This did not tend to be a problem with LAMS.

The Geometry of LAMS compared to SEMS makes them an attractive option for management of post-operative strictures in patients who have undergone RYGB. The dumb bell shape allows the stent to remain relatively fixed across a short stricture. In our experience LAMS was still prone to migration with a 19% migration rate. The advantage of using the LAMS was that none of these patients required either endoscopic or surgical removal unlike migration of a traditional SEMS. These often require laparoscopy and enterotomy to remove.

LAMS progressively dilate over a 48–72-h time frame and can safely remain in for up to 106 days in our experience. This allows a slow steady radial pressure to be applied to the stricture. We believe this results in a more permanent outcome. Pneumatic dilatation may not apply enough radial force to the strictured segment of anastomosis to give a sustained result.

In our experience we found early removal (< 40 days) resulted in an increased risk of re stricture of the gastrojejunal anastomosis. Others have found up to 100 days median time for resolution of Gastric strictures [18], we propose initial placement for a minimum of 40 days before removal. It is unclear whether initial stricture diameter is a factor yet, but it is possible that the smaller the diameter of the stricture may have an impact in stricture resolution? Patients tolerated the LAMS device well and provided there was no proximal migration or placement over the gastro esophageal junction (GEJ) there were rarely symptoms and the device were tolerated well.

A variety of complication’s have been described after placement of LAMS and SEMS. Yang et al. [18] reported a post procedural bleed requiring 2 units of blood after deploying a LAMS across a gastric stricture. We did not experience bleeding or ulceration associated with LAMS placement.

Proximal migration into the distal esophagus did occur in one patient with a short pouch. The patient complained of pain and reflux. On repeat endoscopy the stent was angulated and had not completely deployed. Simple removal and replacement of the stent resulted in a good outcome.

Another role may be in achieving adequate nutritional status prior to more definitive surgical treatment in patients with refractory strictures. This may limit the need to place naso jejunal feeding tubes or feeding gastrostomy prior to formal surgical revision. These are often poorly tolerated and carry significant morbidity of their own.

## Conclusion

Gastro jejunostomy strictures are relatively uncommon after RYGB. They present with dysphagia, nausea, abdominal pain and vomiting [6]. Some patients present with weight loss and hypoalbuminemia. In this retrospective study it appears LAMS may have a role in management of gastro jejunostomy strictures after RYGB.

Their safety profile appears satisfactory with no immediate adverse outcomes. Distal migration remains relatively high but does not appear to adversely affect the patient. Proximal migration can be an issue with short pouches and proximity of the gastro jejunostomy to the GEJ. Distal migration does not appear to require further intervention to remove them.

Migration distally does not tend to result in impaction within the Ileo caecal valve or small bowel meaning the risk of Small Bowel obstruction secondary to stent impaction appears minimal.

A number of other uses are also possible including strictures related to VBG and the potential role in small leaks after RYGB, especially if they are associated with a stricture.

The procedure can be successfully performed without radiology and can be done as an outpatient. LAMS appear to be effective in preventing multiple procedures for resolution of strictures and increase rates of stricture resolution compared to dilatation alone.

## Data Availability

The data supporting our findings are available in this article. The raw data sheets are not publicly available. Articles referred to can be found in the reference list.

## Abbreviations

BMI: Body mass index
RYGB: Roux-en-Y Gastric Bypass
LAMS: Lumen apposing metal stents
SEMS: Self expanding metal stents
